# Illness coping experiences among patients receiving peritoneal dialysis: a descriptive phenomenological study

**DOI:** 10.3389/fpsyg.2026.1924070

**Published:** 2026-10-28

**Authors:** Huixing Huang, Leilei Chen, Guozhi Liu, Xiquan Lu, Rongrong Wang, Yanhua Tian

**Affiliations:** 1 The Second Clinical College of Guangzhou University of Chinese Medicine, Guangzhou, Guangdong, China; 2 School of Nursing, Guangzhou University of Chinese Medicine, Guangzhou, Guangdong, China; 3 Zhongshan Hospital of Traditional Chinese Medicine, Zhongshan, Guangdong, China; 4 Zhongshan Hospital of Traditional Chinese Medicine Affiliated to Guangzhou University of Chinese Medicine, Zhongshan, Guangdong, China; 5 The Second Affiliated Hospital of Guangzhou University of Chinese Medicine, Guangzhou, Guangdong, China

**Keywords:** coping strategies, illness challenges, illness experiences, patients receiving peritoneal dialysis, qualitative study

## Abstract

**Aims:**

To explore the illness coping experiences of Chinese patients receiving peritoneal dialysis and to provide directions for interventions that promote the integration of treatment into daily life and improve quality of life.

**Design:**

A descriptive phenomenological study.

**Methods:**

We conducted semi-structured interviews with 14 patients receiving peritoneal dialysis between 1 August and 30 December 2025. Participants were recruited using maximum variation purposive sampling from the nephrology department of a tertiary hospital in Guangdong Province. Data were analyzed using Colaizzi’s seven-step phenomenological method.

**Results:**

Three core themes were identified: illness experiences (concealment of illness, distress from physical manifestations, and complex psychological reactions), illness challenges (rigidification of daily routines, restricted mobility, dietary limitations, and need for a clean environment), and coping strategies (support from family and friends, healthcare professional support, and self-transcendence).

**Conclusion:**

Coping with peritoneal dialysis is multidimensional, involving concealment of illness, physical distress, psychological reactions, daily-routine challenges, and coexisting coping resources. Clinical nursing needs to move from symptom management toward holistic care, strengthen family and professional support, and support patients in maintaining meaning and social connection.

## 1 Introduction

Epidemiological data indicate that the global prevalence of chronic kidney disease (CKD) is approximately 13.4% (Lv and Zhang, 2019). In China, an estimated 132 million individuals are living with CKD, representing nearly one-fifth of the worldwide total (GBD CKDC, 2020). As the CKD population continues to grow, the number of patients progressing to end-stage renal disease (ESRD) has also risen. The primary treatment modalities for ESRD include kidney transplantation, hemodialysis (HD), and peritoneal dialysis (PD). Among these, PD is utilized by approximately 11% of the global dialysis population due to its relatively lower treatment costs and greater convenience for daily living, occupying a significant role in renal replacement therapy (Himmelfarb et al., 2020). PD is a home-based renal replacement therapy that uses the patient’s peritoneum as a semi-permeable membrane to remove excess water and metabolic waste products (Gokal and Mallick, 1999).

While PD is effective in clearing uraemic toxins and alleviating uraemic symptoms, as a lifelong and invasive replacement therapy it also imposes a substantial treatment-related burden. Physiologically, patients must adhere to stringent fluid restrictions and complex self-management procedures, and they are at risk of catheter-associated infections, progressive decline in peritoneal membrane function, malnutrition, and fatigue (Bonomini et al., 2024; Cho et al., 2024; Guedes et al., 2022; Han and Han, 2012). These burdens may impair functional capacity and health-related quality of life. Psychologically, individuals receiving PD frequently experience anxiety, depressive symptoms, treatment-related burnout, and body image disturbance, which may reinforce one another and reduce quality of life (Chan et al., 2019; Griva et al., 2010; Griva et al., 2014; Jung et al., 2019; Marki et al., 2023).

A distinctive illness experience is generated among patients receiving peritoneal dialysis by multiple contributing factors. These factors are identified as treatment-induced alterations in body image, the presence of an indwelling catheter, and the constraints imposed by a home-based therapeutic environment. Concurrently, daily life and psychosocial adaptation are continuously challenged. These challenges arise from performing dialysis exchanges three to five times daily, adhering to stringent fluid and dietary regimens, and sustaining a high-intensity self-care workload (Yang et al., 2024). Consequently, the systematic investigation of illness experiences and coping strategies in patients receiving peritoneal dialysis has been recognized as a critical research priority. This investigation is required for advancing the quality of care and improving clinical outcomes in this population.

Illness experience constitutes an inseparable, inherent component of the disease, and clinical practice must therefore fully recognize and systematically address it (Madeira et al., 2019). As a related construct, coping experience refers to the cognitive, emotional, and behavioral responses that an individual adopts under conditions of stress and challenge. These responses function to regulate emotion and solve problems. Concurrently, genuine feelings and psychological experiences emerge during this process, forming an integral part of the coping experience (Folkman and Moskowitz, 2004). In the existing literature, scholars have sought to integrate illness experience and coping experience. Some have tentatively proposed a summative definition of “illness coping experience.” This definition preliminarily characterizes illness coping experience as a dynamic and integrated cognitive, emotional, and behavioral process. Following the onset of illness, an individual initiates this process to cope with disease-related stress. More specifically, the process encompasses the individual’s perception and understanding of the illness, along with the subsequent adoption of strategies such as support seeking, emotion regulation, and lifestyle modification (Wu et al., 2026). Contemporary medical research and clinical practice have increasingly directed attention toward patients’ subjective experiences and the coping strategies they employ during illness adaptation. This trend is evident across multiple fields, including mental disorders (Sandhu et al., 2013), chronic diseases (Zugaj et al., 2025), and cancer (Xia et al., 2024). Previous qualitative research in dialysis has focused mainly on symptom burden, quality of life, and self-management. However, qualitative evidence on how Chinese patients receiving PD experience illness and cope within their sociocultural context remains limited. In China, illness disclosure, stigma, family responsibility, and family-centered support may shape how patients interpret and manage illness. This study therefore aimed to explore the illness coping experiences of Chinese patients receiving PD using a descriptive phenomenological approach.

### 1.1 What does this paper contribute to the wider global clinical community?

Clinical practice should go beyond symptom management to include stigma reduction, family-centered support, and peer-sharing interventions, helping patients sustain meaning and social connection while living with PD.

This study aimed to address the limited qualitative evidence on the illness experiences and coping strategies of Chinese patients receiving peritoneal dialysis within a non-Western sociocultural context. The findings reveal how peritoneal dialysis constrains patients’ temporal autonomy, spatial mobility, and daily rhythms. These insights can inform holistic care models for other long-term home-based therapies globally, such as home hemodialysis and parenteral nutrition, thereby enriching cultural diversity in the existing literature.

## 2 Methods

### 2.1 Design

This study employed a descriptive phenomenological approach. It aimed to explore the illness experiences and coping processes of patients receiving PD. Interviews were conducted by a nursing postgraduate researcher with approximately 6 months of qualitative research experience, who had no prior relationship with participants. We collected data through face-to-face semi-structured interviews and interview field notes, and analyzed the data systematically using Colaizzi’s seven-step phenomenological method. Bracketing was used as an ongoing reflexive process to identify and manage researchers’ pre-understandings. The reporting of this study was guided by the Consolidated Criteria for Reporting Qualitative Research (COREQ) guidelines (Tong et al., 2007), and a completed COREQ checklist is provided as Supplementary Material 1.

### 2.2 Study setting and recruitment

This study was conducted between 1 August and 30 December 2025 in the nephrology department of a tertiary hospital in Guangdong Province. Participant recruitment followed the maximum variation principle within purposive sampling. This strategy ensured sufficient diversity across the dimensions of sex, age, place of residence, and duration of PD treatment. Sample size was determined according to the principle of data saturation (Francis et al., 2010). Saturation was first discussed after the 12th interview, when preliminary subthemes appeared stable. Two researchers jointly reviewed the emerging codes, meaning units, subthemes, and themes. After the 14th interview, no new subthemes or higher-order themes emerged. Saturation was therefore defined at the level of subthemes and themes, rather than as a purely numerical endpoint. Ultimately, a total of 14 patients receiving peritoneal dialysis were interviewed.

Potential participants were initially identified through a review of medical records and through consultation with the physicians and nurses responsible for their care. To minimize potential gatekeeping, these clinicians initially introduced the study, but the research team independently confirmed eligibility, explained voluntariness, and assured participants that non-participation would not affect their care. Written informed consent was obtained from all participants.

Inclusion and exclusion criteria were as follows:

Inclusion criteria: (1) currently receiving regular peritoneal dialysis treatment; (2) dialysis duration ≥ 3 months; (3) age ≥ 18 years; (4) alert and able to complete the survey independently in written or oral form.

Exclusion criteria: (1) patients with communication barriers or impaired consciousness; (2) patients with other severe comorbid conditions, operationally defined as acute infection, decompensated heart failure, terminal malignancy, severe cognitive impairment, or any condition that prevented sustained conversation; (3) patients with active severe psychiatric symptoms that prevented informed consent or participation in an interview. This criterion was applied only when psychiatric symptoms were active and severe enough to impair informed consent or interview participation; patients with stable or historical psychiatric conditions were not excluded. (4) Patients with previous hemodialysis or kidney transplantation. These criteria were applied throughout the recruitment period.

It should be noted that this study employed a single in-depth interview to complete data collection. No intervention or follow-up was involved. The issues of sample “elimination” or “dropout” were therefore not applicable. Data from all participants who completed the interview were included in the final analysis.

### 2.3 Data collection

We collected data using one-on-one, semi-structured interviews. The interview guide was developed through consultation among the team’s experts and was pilot-tested in two preliminary interviews with participants N1 and N2. These two preliminary interviews were included in the final sample because the interview guide was considered appropriate and the data were sufficiently rich. No changes were made to the interview guide after these preliminary interviews; the preliminary interviews confirmed the clarity of the questions, the appropriateness of the interview duration, and the feasibility of the interview procedure. The final interview guide is presented in Table 1. The researcher arranged each interview at a time that suited the participant. The locations chosen were quiet, private, independent spaces, such as a departmental teaching room or a conference room. Before each interview, the researcher restated the study purpose and content to the participant and reaffirmed the principle of confidentiality. Informed consent was then obtained. Once consent had been granted, the researcher audio-recorded the interviews to capture verbal data, including speech hesitations and emotional shifts in tone. Non-verbal signals observed during the interviews (e.g., body movements and facial expressions) and contextual information were documented by the interviewer in field notes. Each interview lasted for a minimum of 30 min. After each interview, the researcher transcribed the audio recording verbatim within 24 h. All personally identifiable information was then removed from the transcript. During the same period, we maintained a reflexive journal, documenting interview feelings, preliminary analytic insights, and the potential influence of the research process on the researcher. All interviews were completed in a single session. No supplementary interviews were required, as the initial interviews provided sufficient information for analysis. All interviews were conducted in Mandarin Chinese or Cantonese, according to the language preferred by each participant. The audio recordings were transcribed verbatim in Chinese, and initial coding and theme development were also conducted in Chinese to preserve the original meanings of participants’ accounts. Participant quotations were translated into English at the manuscript preparation stage, after data analysis had been completed. The translation was performed by the third author, and the translated quotations were independently checked by the two co-first authors. All translated quotations were back-translated to check for semantic equivalence with the original Chinese transcripts.

**Table 1:** Outline of the interview.

Questions
1. Can you share with me your experience of undergoing peritoneal dialysis?
2. What was your initial reaction when you first learned that peritoneal dialysis would be necessary for you?
3. How has peritoneal dialysis affected your daily life? What changes has it brought to your life overall?
4. What difficulties have you encountered, both in performing the peritoneal dialysis procedures and in managing your dietary restrictions?
5. Since starting peritoneal dialysis, have you experienced any emotional fluctuations? How have you managed to cope with these feelings?
6. In the process of managing your illness, what feelings or personal insights have you gained?
7. Is there anything else you would like to share about your life after starting peritoneal dialysis?

The interview guide was used flexibly. Open-ended probes were used to explore unexpected experiences, including “Can you tell me more about that?,” “What was that like for you?,” “Could you give an example?,” and “How did that affect your daily life?” The final themes were derived inductively from participants’ accounts.

### 2.4 Data analysis

Data collection and analysis were conducted concurrently. The interviewer transcribed each recording verbatim, and another researcher cross-checked the transcripts against the audio recordings to minimize information loss. This study followed a descriptive phenomenological approach. Bracketing was conceptualized not as the complete elimination of presuppositions, but as a systematic process of identifying, documenting, and critically examining researchers’ pre-understandings, assumptions, clinical knowledge, and expectations (Gearing, 2004). Before data collection, the team reflected on assumptions that patients receiving PD might experience stigma, family burden, and loss of meaning. During analysis, researchers repeatedly returned to participants’ original words, kept reflexive notes, and discussed alternative interpretations in team meetings. Consistent with this approach, Colaizzi’s seven-step method was used to analyze the data and identify themes (Edward and Welch, 2011). The specific steps were as follows: (1) the interview transcripts and field notes were read repeatedly to understand the participants’ accounts. Field notes were used as supplementary contextual data to support understanding of participants’ accounts and were not analyzed as an independent data source. (2) Significant statements were extracted and coded. (3) Similar codes were grouped to generate initial concepts. (4) These concepts were linked back to the raw data and elaborated in detail. (5) The concepts were organized and refined into themes and subthemes. (6) The themes were verified against the original data and refined accordingly. (7) Member checking was conducted with three participants; one responded, reviewed a summary of the preliminary themes, and indicated that no changes were needed. Because only one participant provided feedback, the final themes were also verified by the research team against the original transcripts. All transcripts were jointly coded by two researchers, rather than only a subset. Coding disagreements were first discussed between these two researchers. When consensus could not be reached, the issue was brought to the wider author group for discussion until consensus was achieved. After preliminary themes were generated, the wider author group reviewed them against the original transcripts before final themes were confirmed by all authors. An illustrative example of the analytic progression from raw quotation to significant statement, code, subtheme, and final theme is provided in Supplementary Material 2.

### 2.5 Ethical considerations

This study was approved by the Ethics Committee of Guangdong Provincial Hospital of Chinese Medicine (Approval No.: ZM2025-301; Approval Date: 23 July 2025). The study was conducted in strict accordance with the ethical principles of the Declaration of Helsinki. All participants were informed about the research content and signed informed consent forms. Participation was entirely voluntary. Given the sensitive nature of the interviews, participants’ psychological safety was protected by the following measure: before each interview, the researcher informed participants that they could decline to answer any question that made them feel uncomfortable or stop the interview at any time without giving a reason. To protect participant privacy, all interview data were anonymized. Patient names were replaced with numerical codes.

## 3 Findings

A total of 14 patients undergoing peritoneal dialysis were interviewed for this study. The participants consisted of 7 men and 7 women. Their ages ranged from 29 to 63 years, and their dialysis vintage spanned from 5 to 60 months. The demographic characteristics of the participants are presented in Table 2.

**Table 2:** Sociodemographic characteristics of participants (*N* = 14).

No.	Gender	Age	Marital status	Occupation	Educational background	Residence	Time since peritoneal dialysis (months)	PD modality	Complications
N1	Male	54	Married	Teacher	Bachelor’s Degree	Urban Area	36	CAPD	/
N2	Female	43	Married	Teacher	Bachelor’s Degree	Urban Area	24	CAPD	Hyperphosphatemia
N3	Male	54	Married	Unemployed	Junior High School	Rural Area	60	CAPD	Hyperphosphatemia
N4	Female	45	Married	Self-employed	Bachelor’s Degree	Urban Area	24	CAPD	/
N5	Female	36	Married	Company Employee	Senior High School	Urban Area	18	CAPD	/
N6	Female	52	Married	Retired	Bachelor’s Degree	Urban Area	42	CAPD	Calcium-phosphorus Imbalance
N7	Female	62	Married	Unemployed	Junior High School	Urban Area	5	CAPD	/
N8	Male	34	Unmarried	Unemployed	Secondary Vocational Education	Urban Area	23	CAPD	/
N9	Male	29	Married	Freelancer	Bachelor’s Degree	Urban Area	24	CAPD	Secondary Hyperparathyroidism
N10	Female	42	Married	Freelancer	Senior High School	Urban Area	36	CAPD	Peritonitis
N11	Male	57	Married	Government Official	College Degree	Rural Area	8	CAPD	Peritonitis
N12	Female	60	Married	Unemployed	Junior High School	Urban Area	6	CAPD	/
N13	Male	52	Married	Self-employed	Junior High School	Urban Area	28	CAPD	/
N14	Male	63	Married	Unemployed	Junior High School	Rural Area	54	CAPD	Hyperphosphatemia

### 3.1 Themes

The average interview duration was 55 min, with a range of 33 to 110 min, yielding a total transcript volume of approximately 137,985 Chinese characters. Through repeated comparison, categorization, and analysis of the interview data, three core themes were identified: illness experiences (concealment of illness, distress from physical manifestations, and complex psychological reactions), illness challenges (rigidification of daily routines, restricted mobility, dietary limitations, and need for a clean environment), and coping strategies (support from family and friends, healthcare professional support, and self-transcendence) as illustrated in Figure 1.

**Figure 1:**
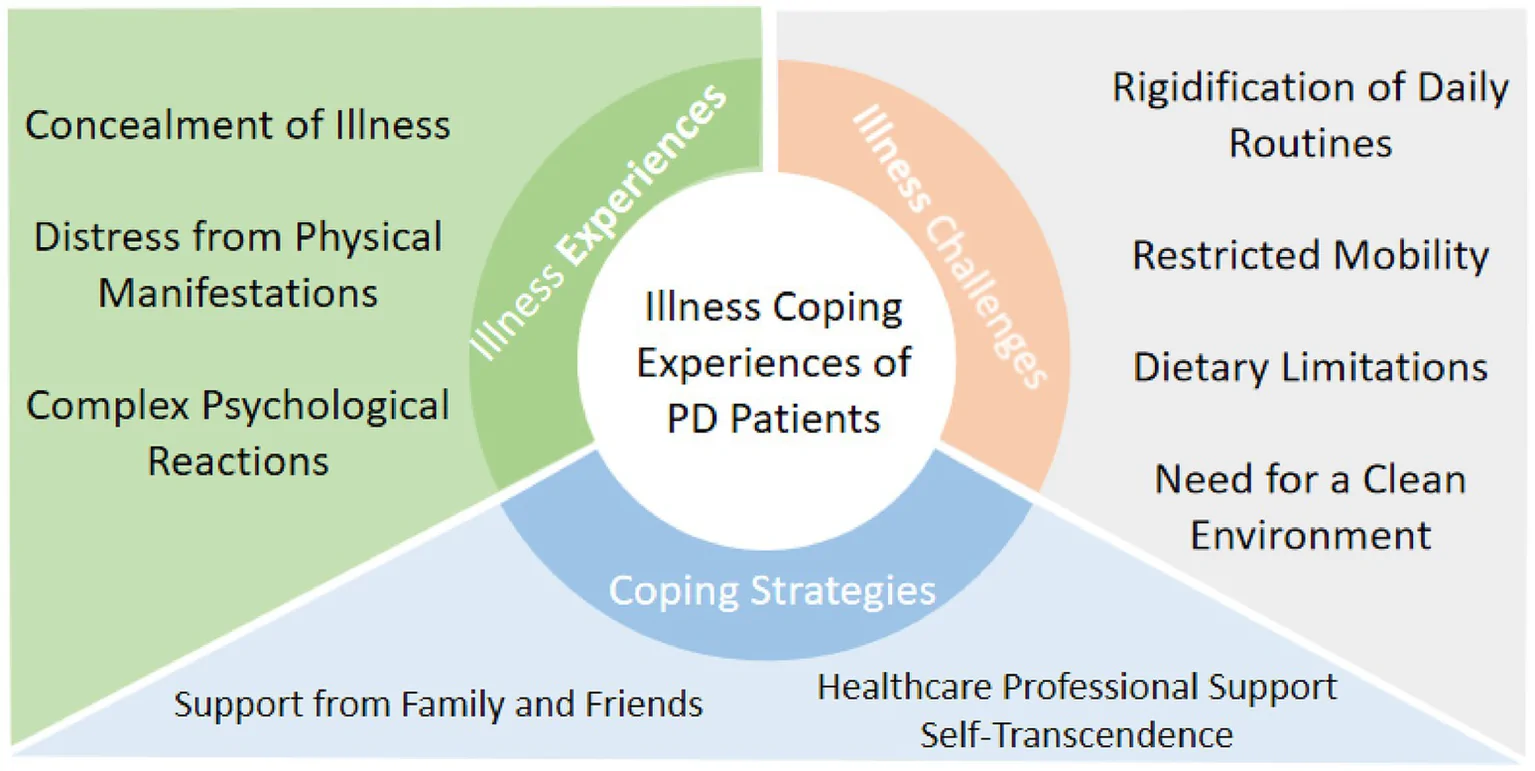
Overview of the three core themes and their respective subthemes identified from the interview data. The three overarching themes are analytic categories representing different dimensions of the illness coping process. Illness experiences refer to subjective perceptions, emotional responses, and meaning-making; illness challenges refer to treatment-related constraints on everyday life, including time, mobility, diet, and environment; coping strategies refer to cognitive, emotional, and behavioral responses, including the mobilization of external resources.

#### 3.1.1 Illness experience

##### 3.1.1.1 Concealment of illness

Following the onset of illness, many participants tended to conceal their condition from others, exercising strict control over disease-related information and communicating it only on a highly selective basis. Typically, only the closest family members and friends were informed, while other social contacts were deliberately excluded. This behavior was driven by a deep-seated fear of being gossiped about, singled out, treated differently, or exposed to stigmatizing glances. Participants commonly perceived PD as a blemish that, in the eyes of others, could invite unfavorable judgment. Once revealed, risk damaging their normal social image. To maintain the appearance of being a normal person, some even adopted specific disguises in daily life to mask the traces of treatment and prevent others from detecting any sign of their condition. Such concealment behavior not only reflected a fear of social stigmatization but also imposed a persistent psychological burden on the participants.

*N2: Apart from my family and closest friends, I basically never told anyone I’m on PD, certainly not people I’m not that close to.*

*N5: Being on PD is not exactly a good thing. I was worried that if other people found out, word would get around. There’d be gossip, people pointing fingers at me. So only my family and a few close friends know. I’ve never brought it up with anyone else.*

*N8: I’ve always felt that I do not really want people to know I have kidney disease and need PD. Because once they know, I might become the focus of their gossip.*

*N13: My family runs a breakfast shop. I had already thought about it beforehand. If the delivered dialysate was spotted by the neighbors when it arrived, I would just tell them it was ingredients for the shop.*

##### 3.1.1.2 Distress from physical manifestations

Distress from physical manifestations constituted a core experience commonly shared by patients receiving peritoneal dialysis, manifesting primarily at two levels: physiological symptom burden and body image disturbance. At the level of physiological symptom burden, patients endured multiple physical discomforts. Abdominal distension arose frequently from the combined influence of dietary intake and dialysate, while declining physical strength and persistent fatigue curtailed their daily activities. Dizziness, nausea, and vomiting further intensified their sense of physical vulnerability. These ongoing physiological symptoms not only impaired their functional capacity but also led to an involuntary contraction of their social life.

*N3: After starting PD, I cannot eat too much, but I cannot skip meals either. The other night I ate a bit more, and my belly felt really bloated. I cannot do much exercise now either which cause not much chance to break a sweat.*

*N9: I was quite thin to begin with. After PD, I often get dizzy, nausea, vomiting. When I’m stressed, I tend to get acid reflux, and that makes me feel like throwing up.*

*N12: It’s not that I do not want to socialize. It’s just that my body feels weaker now, and I get tired easily. I do not feel like going downstairs, because climbing back up the stairs really wears me out. So I’ve gradually stopped hanging out with people as often.*

Meanwhile, some also faced changes in physical appearance induced by dialysis treatment that manifested as body image disturbance. For instance, abdominal distension forced them to alter their dressing habits. Changes such as a sallow complexion, acne, localized skin hyperpigmentation, and an older-looking face led them to appraise their own appearance negatively. These treatment-induced changes fostered negative perceptions of their physical image, which in turn eroded their sense of self and led some patients to withdraw from social situations.

*N4: At first I was really bothered by how PD affected my body shape. My belly got bigger, and none of the clothes in my wardrobe fit me anymore. I could not wear high heels or dresses either, because they got in the way of doing the dialysis.*

*N6: After starting PD, my skin turned really dark, and I looked much older. Everyone said I looked like someone in their seventies. Part of the reason I do not feel like going out and socializing is exactly these changes caused by the treatment.*

*N9: After starting peritoneal dialysis, I started getting pimples, and my complexion became duller than before. So now I’m very conscious of how my appearance has changed.*

##### 3.1.1.3 Complex psychological reactions

As PD treatment became routine, patients faced profound psychological blows and endured persistent worry and uncertainty about the future. Although these reactions were intertwined, two analytically distinct dimensions were evident. The first dimension concerned fear of progression and uncertainty, with concerns about roles and finances interwoven within it. They feared that dialysis could never fully replace renal function and that toxins accumulating in the body would gradually damage other organs. They anxiously monitored fluctuations in their laboratory indicators, interpreting each abnormal result as a signal of physical deterioration. They also worried about the threat the disease posed to their work, family, and life itself, repeatedly asking themselves how much longer they could be with their loved ones and whether they could sustain their former social roles. This fear of disease progression was ever-present, rooted not only in a sense of losing control over the illness itself but also in a deeper uncertainty about the length and quality of their remaining life. It became an inescapable psychological burden that haunted their daily existence.

*N2: I know that poor kidney function affects the removal of toxins and can also damage other organs. So I keep worrying that my health will only get worse and worse.*

*N9: I get anxious about things like — how long can I live with this illness? Can I keep working? Will I be able to hold my marriage together? Will my parents have anyone to depend on?*

*N10: When my PD benefit aren’t particularly good, or when my lab values fluctuate and I lose my appetite, I start worrying that my physical condition is just going downhill.*

*N11: At the very beginning, I felt anxious and panicky. I was afraid because after starting PD, I just did not know what to do or whether I could ever get better.*

A second dimension concerned existential distress and loss of meaning. Some participants reported that the relentless daily routine of peritoneal dialysis, the loss of a normal life, and shattered expectations for the future gradually gave rise to deeper psychological distress. They began to feel that life had lost its meaning and direction, sinking into a state of hopelessness and emptiness.

*N5: At the beginning, I used to cry sometimes in the hospital ward. I just could not accept having to do three bags of dialysis fluid a day that felt like such a hassle.*

*N6: I feel like my life has no rhythm at all. My days revolve around nothing but taking medication and doing dialysis, and I have no one to keep me company. It feels like life is just wasting away. Days like this make me really anxious, because I feel I’ll never live like a normal person again.*

*N9: Ever since I was little, I knew I’d have to rely on myself in the future — build my own family and not end up living in a blended family. But after starting PD, it felt like a dream had shattered. Especially at night, I would cry and keep asking myself: Why did I get sick? Why me? Why did not I take better care of my health before? These questions kept pounding at me.*

*N14: I feel really agitated inside. After starting PD, I’ve had no income, yet the treatment costs money. I also feel that life has become meaningless, as if I’ve lost interest in everything.*

#### 3.1.2 Illness challenges

##### 3.1.2.1 Rigidification of daily routines

The strict timing requirements of PD cut patients’ daily lives into small blocks centered on each dialysis exchange. Their daily schedules were decided by when they needed to perform the exchanges. All activities had to fit around these fixed times. Every trip outside was limited by the gap between two exchanges, and anything that took longer simply could not be done. This rigid time structure took away their sense of control over their own lives. With each passing day, as they repeated the same routines, they gradually felt they were losing the independence and freedom they once had as healthy individuals.

*N3: I have to do at least four or five bags of dialysis fluid every day. It takes up so much time. To put it simply, it’s like I’ve turned into a person who cannot do anything.*

*N7: I have absolutely no desire to go out now, because in the back of my mind, I’m always thinking I need to get home for the dialysis. It’s not like before, when I could stay out as late as I wanted.*

*N12: PD messes up my daily habits, but there’s nothing I can do about it. Now I have to do an exchange every four hours, so I can only manage a short walk around the neighborhood.*

*N13: My whole day is taken over by PD. If I go out, I’ve only got those few hours in between exchanges before I have to rush back home for the next one.*

##### 3.1.2.2 Restricted mobility

Some participants reported that the rigid requirements of PD for both time and equipment turned outings that used to be spontaneous and flexible into something complicated and difficult. Long-distance travel had become an almost impossible undertaking. Even short trips required them to carry sufficient dialysate, insulated containers, and other supplies, and they often relied on family members for transport. They therefore had much less freedom to arrange outings as they wished. At the same time, the physical decline and fatigue caused by the disease itself turned going out into a physical burden. They perceived it more as a strenuous chore than as a leisure activity, making it hard for them to truly relax.

*N1: When my son was little, I used to take him out all the time. Now I still want to take him on trips, but I just cannot. Because I’d have to bring all the PD equipment, and I cannot just pause the treatment.*

*N2: My travel is very restricted now. I cannot just pick a place, buy a ticket, and set off like I used to. Since starting PD, I have not taken any long trips at all. Even short trips now have to be by car with my husband, because we need to bring the dialysate, the insulated containers, and all that gear.*

*N6: Sometimes I’d like to go to a nearby city for a day or two, but even that is kind of difficult for me now. I can only do it if my family drives. And when I look at all that dialysate piled up in the car, I get really annoyed.*

*N9: Before, when I traveled, I could wander around everywhere. But now, even if I do go somewhere, I get tired so easily. There’s no sense of enjoyment — it feels more like a strenuous chore. The physical burden is heavier, and I cannot really relax mentally either.*

##### 3.1.2.3 Dietary limitations

Given the need for strict fluid control and the fear of excessive dietary intake of salt, phosphorus, and potassium, patients frequently found themselves in a dilemma during social dining occasions. On the one hand, they were uncertain which dishes on the table were beneficial or harmful to their condition. On the other hand, they were reluctant to make special requests that might dampen the mood of the gathering. Ultimately, they often chose to reduce or avoid dining out altogether.

*N4: Some dishes on the menu I just cannot eat, and I’m never sure whether restaurant food might be harmful to my condition. So I choose to avoid eating out.*

*N6: Restaurant food tends to have a lot of additives, so I try to eat out as little as possible. Sometimes when I do meet up with friends, I’ll suggest going for dim sum — at least there I can have some steamed buns or dumplings.*

*N13: After starting peritoneal dialysis, I’ve become less willing to keep in touch with friends, and my diet is at least one reason for that. My body will not allow me to go out and eat and drink freely the way they do.*

##### 3.1.2.4 Need for a clean environment

Given that any contamination during PD procedures could trigger serious complications such as peritonitis, patients maintained heightened vigilance over the sanitary conditions of their home environment and its surrounding areas. To minimize infection risk, some set aside a dedicated space at home exclusively for dialysis operations, strictly restricting others from entering this area while adhering to daily disinfection and changing clothes upon entry and exit. Moreover, when eating out, they also worried about germ exposure resulting from improper food handling. Some practices followed standard PD infection-prevention guidance, such as daily disinfection, hand hygiene, and wearing a mask during exchanges. Other accounts reflected heightened or potentially excessive vigilance about contamination, such as restricting all others from entering the dialysis room or persistent worry about germs in restaurants.

*N6: At home, I’ve set aside a room just for doing PD. I will not let anyone else go in there. Every day, I disinfect it. When I come back from outside, I change my clothes and shoes before I go in — just to keep everything fairly clean.*

*N12: The doctor told me that the most important thing with PD is to pay attention to hygiene. So my daughter insists that I must disinfect everything every day.*

*N13: I worry about the hygiene of the restaurant environment and the food. I’m afraid the food might not be cooked well enough, or that the ingredients aren’t handled strictly, and that they could carry germs.*

#### 3.1.3 Coping strategies

##### 3.1.3.1 Support from family and friends

Most participants reported that the understanding and assistance they received from family members helped motivate them to confront the disease with a more positive outlook and to adjust their mindset accordingly.

*N4: My family does a lot of things related to PD for me, like the disinfecting. My child has also started to take on real responsibilities, so many things no longer need me to worry about them.*

*N9: My wife has always stood firmly by my side. She made me feel that I really should pull myself together, face this bravely, and take responsibility for myself and for the people who truly care about me. It was exactly because of my family’s encouragement and their constant presence that I slowly began to pull out of those thoughts of life was meaningless and that I did not want to go on living.*

*N10: Every time something is off with me, my husband can sense it. Whenever I tell him I’m not feeling well, I cannot eat, or I’m feeling down, he just puts his arms around me. That helps me so much.*

*N13: My family said, no matter how much it costs, we’ll get treatment. When I got sick, my brother-in-law immediately said he would take full responsibility for all my medical expenses.*

Some participants indicated that, beyond family, support from friends also played an important role in coping with the disease. Such emotional comfort and social connectedness helped them gradually return to a normal life.

*N6: When I first started PD, I took sick leave and stayed home. My colleague came by every evening to take a walk with me. I felt that I was still needed — needed by others, needed by society, needed by my family. That was the very reason I could accept, so quickly, that I needed treatment for life.*

*N2: After my best friend learned that I needed peritoneal dialysis, our conversations started to revolve more around her concern for me. She also began looking up information and treatment-related materials to help me out.*

##### 3.1.3.2 Healthcare professional support

The personal warmth and patient guidance offered by healthcare professionals not only helped patients become proficient in peritoneal dialysis procedures but also provided them with emotional comfort at a psychological level. Some patients established trusting relationships with their healthcare providers. In these interactions, they felt cared for and accepted, and this support became an important psychological anchor in coping with the disease.

*N8: Being treated here, I feel that the healthcare staff are all very warm. It’s more like an emotional connection, not just them doing their job.*

*N9: The first time I came to the hospital to change my dressing, the nurse who guided me was really gentle. That helped me relax a lot, and I wasn’t as scared or anxious anymore.*

*N10: Here I feel so much care that it gives me a sense of being at home. Sometimes I can just be myself and let them pamper me a bit, and we get along just like friends.*

*N14: I usually come to the hospital by myself. The healthcare staff give me timely help and talk with me, which really lifts my mood.*

##### 3.1.3.3 Self-transcendence

Some participants moved beyond their own suffering by reconnecting with others and finding purpose beyond themselves. They found strength in being needed and in taking responsibility for others.

*N6*: After starting PD, I took a part-time job at an after-school care center, checking the children’s homework. The children relied on me. Once I arrived a little late, and they all rushed over and asked, ‘Teacher, why are you so late? I thought you were not coming’. At that moment, I truly felt that I was still needed, and I felt more fulfilled.

*N9:* I felt that I should stand up, face it bravely, and be responsible for myself and for the people who truly care about me.

For some participants, this shift also involved re-evaluating what mattered in life and rebuilding a sense of meaning after illness.

*N2: Lately I’ve been learning to write calligraphy with a brush by following online videos. I might spend several hours a day practising, and that makes me feel that life is not quite so meaningless after all.*

*N4: Now that my time and energy have come back to myself, I see my relationships with others more rationally.*

Together, these accounts illustrate how some participants moved beyond self-focused distress through being needed by others, taking responsibility for them, and reconstructing meaning and value in life.

## 4 Discussion

### 4.1 Complexity of illness experience involves negative psychological distress and adverse physical perceptions

In interpreting these findings, we draw on existing theoretical literature as interpretive lenses while remaining close to participants’ accounts. Participants reported treatment-related physical discomfort and body image disturbance. They often linked these experiences to fear of judgment and uncertainty about the future. These accounts suggest that physical changes and psychological distress may be closely intertwined. Concurrently, the absorption of high-concentration glucose from the dialysate may increase caloric intake and contribute to metabolic dysregulation, while prolonged dialysis may accelerate muscle protein catabolism. Together, these mechanisms may contribute to energy deficiency, malnutrition, and other physical complications (Wilkinson et al., 2021). Simultaneously, the dialysate continuously dwelling in the peritoneal cavity compresses the gastrointestinal tract. This elevates intra-abdominal pressure and consequently induces digestive symptoms such as gastro-esophageal reflux, dyspepsia, and constipation (Yi et al., 2022). In addition, patients must perform daily PD exchanges, and the persistent physical load imposed by the dialysate permanently retained in the abdominal cavity may contribute to abdominal distension. They also reported physical discomfort as closely tied to psychological irritability and body image concerns. These experiences appeared to heighten their fear of illness exposure. For instance, most participants disclosed their condition only to their closest family members, and some even adopted specific disguises to conceal the traces of treatment. Although such behavior may partly reflect a desire to protect privacy, participants often described a deep-seated fear of disease-related stigma as a key concern. A key factor is that PD, as a lifelong replacement therapy, involves an indwelling catheter, frequent dialysate exchanges, and treatment-induced bodily changes—all of which are highly susceptible to social labeling.

Drawing on Goffman’s concept of a spoiled identity (Goffman, 2009), these accounts may be interpreted as reflecting concerns about social judgment. Existing evidence indicates that stigma is associated with poorer physical and mental health, lower quality of life and hope, as well as diminished self-esteem and self-efficacy (Ali et al., 2012; Diao et al., 2025; Livingston and Boyd, 2010; Malli et al., 2016). Some spoke of withdrawing from social contact after anticipating judgment. This may be understood as a process through which perceived stigma and social withdrawal were closely connected. In a healthy state, the body integrates naturally into social life by virtue of its normal functioning and escapes particular notice. This is referred to as absent presence (Shilling, 2021). However, participants described the indwelling catheter, frequent procedures, abdominal distension, and skin changes as making their body a focus of others’ attention, which they linked to feelings of shame and avoidance behaviors. Bodily visibility was described as a persistent concern, and these treatment-related changes were perceived as difficult to reverse. This may be interpreted through the lens of spoiled identity.

Accounts of alienation were also evident, in which the body served simultaneously as the instrument of treatment and the source of suffering. At the physiological level, peritoneal dialysis requires patients to utilize their own peritoneum as a semi-permeable membrane, which participants described as reducing the body to a functional component of medical apparatus. They perceived their body as both a site of treatment and a source of discomfort. This may be understood through the lens of bodily alienation. When symptoms such as abdominal distension and fatigue persisted, they spoke of the body as a reminder of illness, which they said made it difficult to return to their pre-illness self-concept. Research indicates that such persistent fatigue symptoms affect 42 to 89% of dialysis patients and can lead to significant social, psychological, and physical disability (Bello et al., 2022; Yngman-Uhlin et al., 2019). At the level of physical appearance, participants linked outward changes to an intensified sense of alienation. Participants attributed changes such as abdominal distension, sallow complexion, and older-looking appearance to dialysis treatment. Clinically, these changes may also be related to advanced kidney disease, anemia, nutritional status, metabolic abnormalities, treatment effects, or a combination of these factors. Therefore, they should not be attributed solely to treatment (Lev-Wiesel et al., 2022). Research suggests that individuals with body image disturbance often exhibit low self-concept, feelings of worthlessness, and higher levels of stress and depression (Hossain et al., 2020; Marki et al., 2023). These accounts suggest that distress from physical manifestations may extend beyond symptom burden and affect participants’ sense of self.

At its core, fear of disease progression may be interpreted as a projection of death anxiety and concerns about becoming a burden (Sharpe et al., 2023). Participants described their expectations of the future as circumscribed by the disease. Specifically, they described difficulty making long-term life plans in the way that healthy individuals do, and they tended to interpret many bodily signals as harbingers of deterioration, with each fluctuation in their laboratory indicators potentially triggering a recalculation of how much time they may have left. Participants described the peritoneal catheter as a constant reminder of possible worsening illness, which they linked to a pervasive sense of uncertainty. This feature of uncertainty resonates with the psychological experiences of cancer survivors documented by Tan et al. (2021), who found that the mere presence of the disease rendered both groups unable to free themselves from catastrophic expectations about the future. Unlike cancer survivors, participants described this fear as continuous rather than episodic, constantly reactivated by the multiple daily dialysis exchanges. Furthermore, when patients’ daily lives are reduced to a repetitive cycle of dialysis, eating, and resting, and when their social roles are stripped away, leaving them only the identity of a patient, they lose the very possibility of experiencing the fullness of life. Some spoke of the future as uncertain and the present as dominated by treatment. They spoke of life losing meaning and direction. This may be interpreted as existential distress in the dimension of meaning (Bolton et al., 2022; Silva et al., 2024). These accounts suggest that fear may gradually develop into a sense of meaninglessness.

In summary, three interrelated processes were described within illness experience: the social visibility of the body, which participants linked to concerns about identity; the experience of the body as both an instrument of treatment and a source of suffering, which may be interpreted as bodily alienation; and the perception of an uncertain future and a present dominated by treatment, which participants linked to existential distress.

### 4.2 Full spectrum of illness challenges as a restructuring of time, space, and relationships

Participants reported challenges spanning time, space, and social relationships. They described efforts to maintain health and social roles while retaining a sense of control (Kalantar-Zadeh et al., 2021). However, the frequency of PD treatment and the risk of infection were perceived as constraining their capacity to work, travel, or engage in leisure activities.

In the temporal dimension, participants spoke of their daily lives as fragmented into strict time blocks determined by the schedule of dialysis exchanges. This may be interpreted through the concept of disrupted inner time consciousness (Gomersall and Madill, 2015). They reported difficulty allocating time according to their own preferences and felt they had to compromise with the demands of treatment. Time was experienced as fragmented and cyclic, with each exchange resetting their focus on the present and on basic survival maintenance. Some noted a diminished capacity to envisage the future, which they saw as shrinking to the next exchange or the next follow-up visit. They also mentioned difficulty transcending the daily regimen and engaging in proactive life planning, which may be interpreted as a loss of an open future. Research on hemodialysis suggests that the cyclical nature of dialysis may erode temporal autonomy and reduce control over life rhythm, with potential implications for quality of life and psychological well-being (Cluley et al., 2024). PD-specific evidence remains limited, but present findings suggest similar temporal disruptions.

In the spatial dimension, participants reported two sets of constraints related to equipment dependence and environmental cleanliness. On one hand, they perceived their daily activity radius as confined to areas where dialysis equipment remained accessible. Even when travel was possible, they spoke of having to carry sufficient dialysate, insulated containers, and disinfection supplies. This need made travel a meticulously planned journey rather than a relaxing leisure trip. On the other hand, concerns about the cleanliness of external environments made it difficult for them to relax and integrate, even in public spaces such as restaurants. They felt compelled to continuously assess the hygiene conditions of their surroundings and remain vigilant against potential infection risks. This state of heightened alertness may create a psychological barrier between them and the external environment. The tension between spatial accessibility and safety may reinforce tendencies toward social withdrawal and self-isolation. As daily living space became progressively compressed and access to the outside world diminished, participants reported reduced opportunities for social participation and a weakened sense of self-existence.

In the dimension of social relationships, participants experienced a sense of reduced social participation. On one hand, the constraints of time and space made it difficult for them to sustain the frequency and depth of previous social interactions, which they felt pushed them to the periphery of their original social circles. On the other hand, some participants reported proactively reducing social engagement, which may be interpreted as voluntary social withdrawal. This reduced social participation, as expressed by participants, appeared to erode the social roles and identity they held prior to illness, rendering their social support increasingly dependent on core family members (Cai et al., 2024). Research suggests that the narrowing of social support networks and the contraction of social ties may be associated with depressive symptoms, declining treatment adherence, and diminished quality of life (Bello et al., 2022; Liu et al., 2025).

In summary, participants reported illness challenges across three interconnected dimensions of daily life: time, space, and social relationships. These dimensions were seen as mutually reinforcing. Temporal fragmentation was perceived as limiting autonomous control over life rhythm; spatial compression was experienced as restricting possibilities for social participation; and marginalization was felt to further destabilize identity and emotional security. Together, these processes were interpreted as creating a structural predicament in which participants felt their personal agency was progressively eroded and their pre-illness life difficult to reclaim.

### 4.3 Multidimensional coping strategies involving coexisting external resources and internal strengths

This study suggests that the coping strategies described by patients receiving PD were multidimensional, involving both external resources and internal strengths. Specifically, these strategies included support from family and healthcare professionals, alongside self-transcendence and meaning reconstruction. This multidimensional pattern may be interpreted as participants’ efforts to rebuild order in their lives and sustain a sense of self-worth amid the restructuring of body, time, and social relationships (see Findings).

Support from family and healthcare professionals was described as an important resource in coping with the disease. At the family level, support included practical assistance, such as help with disinfection and preparation of supplies, as well as emotional support. For instance, participant N9 described how his wife’s steadfast support helped him move from despair—when he had felt he “no longer wanted to live”—toward facing things bravely. Participant N10 described emotional release and comfort through her husband’s embrace and attentive listening. This acceptance and companionship appeared to alleviate the loneliness and helplessness associated with the disease. This may be interpreted through the stress-buffering model of social support theory (Cohen and Wills, 1985), which proposes that social support may mitigate the negative impact of stressful events by strengthening coping capacity and emotional stability. For patients receiving PD whose social circles had contracted due to treatment, the intensity and quality of family support were described as influencing psychological adjustment. At the healthcare professional level, a safe therapeutic environment was described as being fostered through the clinician–patient relationship, providing participants with a sense of security and trust. This enabled them to receive both professional assistance and emotional comfort when confronting complex self-care procedures and uncertainty. As participant N8 remarked, the warmth of healthcare staff and their communication about his condition made him feel that it was “not just simply about getting the job done.” Such humanistic care, which goes beyond technical competence, may help reduce anxiety in the initial stages and enhance self-management efficacy. It may therefore constitute a critical element in maintaining a trusting relationship between patients and professional treatment.

In this study, self-transcendence refers to expanding concerns beyond the self through being needed, responsibility for others, and a broader sense of connection (Reed, 1991). Adaptation refers to adjusting to illness-related changes, and post-traumatic growth involves positive psychological change following adversity (Tedeschi, 2023). Meaning reconstruction refers to rebuilding a sense of meaning and purpose after disruption (Park, 2010). Although these processes overlap, they are not identical. In this study, self-transcendence is used specifically to capture the relational and existential shift beyond self-focused distress.

Unlike coping strategies that rely on external resources, self-transcendence and meaning reconstruction represent processes in which patients actively restructure their cognitive frameworks and behavioral patterns. Through these processes, they may rediscover meaning in life and a sense of self-worth within the constraints imposed by the disease. This may be interpreted through post-traumatic growth theory (Tedeschi, 2023) and meaning-making theory (Park, 2010) in the psychological literature. Both theories propose that individuals, in struggling with major life crises, may develop positive psychological changes that transcend previous levels of adaptation. In the present study, self-transcendence and meaning reconstruction were described primarily across two interrelated dimensions: behavioral restructuring and cognitive reappraisal. At the level of behavioral restructuring, participants described actively seeking new activities to fill time previously occupied by treatment. For example, some participants channeled time that might otherwise have been consumed by anxiety or emptiness into activities such as calligraphy and reading. This behavioral restructuring appeared to help participants regain a sense of control over their time. As discussed earlier, PD fragments daily life into exchange-centered time blocks, which participants described as limiting autonomy over their life rhythm. When participants actively chose and committed to a meaningful activity, they described reclaiming a space for the self within time that had been appropriated by treatment. This regained control over time may enable a shift from passively accepting treatment arrangements to actively engaging in life planning, allowing participants to gradually rebuild a sense of self-efficacy within the daily treatment cycle. At the level of cognitive reappraisal, the reduction in social roles and social networks created an opportunity for participants to re-examine the nature of interpersonal relationships and core values of life. This cognitive shift may reflect a reconfiguration of value orientation. In health, behavior may be driven by social expectations and external evaluation. In illness, participants described being compelled to confront mortality and rebuild self-identity on a more fundamental level.

However, self-transcendence was not described as an ideal state attainable by all participants. Some participants described external support as enabling inner exploration and meaning reconstruction, while others drew on internal resources.

### 4.4 Cultural context of illness coping

The findings should also be understood within the Chinese sociocultural context. Some experiences described by participants, such as anticipated stigma, concern about becoming a burden, and reduced social participation, have also been reported among PD populations internationally. However, the ways in which these concerns were expressed appeared to be shaped by local sociocultural values. Concerns about illness disclosure were often linked to face-related concerns and fear of gossip, and participants described efforts to maintain a normal social image (Xiong and An, 2025). Family responsibility was evident in participants’ accounts, with some describing themselves as the family’s main provider and expressing a strong obligation not to become a burden. Social identity was closely tied to family roles, and support-seeking was directed primarily toward core family members rather than wider social networks (Diao et al., 2025). Coping was described as family-centered, and meaning reconstruction often involved maintaining family roles, being needed by others, and preserving a sense of normal life. By contrast, temporal fragmentation, dietary restrictions, travel constraints, and infection-prevention practices appeared more directly linked to PD treatment itself and may be shared across international PD populations.

### 4.5 Strengths and limitations

This descriptive phenomenological study adds to the limited qualitative evidence on the illness experiences and coping strategies of patients receiving peritoneal dialysis in China, and provides insight into the diverse ways in which these patients described coping with PD. Our findings offer important directions for shifting clinical nursing practice from traditional symptom management toward holistic care that addresses patients’ psychosocial needs.

However, several limitations should be acknowledged. First, this study was conducted exclusively within a single hospital in Guangdong Province, with all participants recruited from the same medical center. Recruitment was mediated by treating physicians or nurses, which may have introduced gatekeeping or provider-mediated selection bias, as patients perceived as more stable, cooperative, or satisfied with care may have been more likely to be approached. Geographic, institutional, and selection biases may therefore exist, and the transferability of the findings is limited. Second, patients with active severe psychiatric symptoms that prevented informed consent or interview participation were excluded. Although this criterion was considered necessary to ensure informed consent and participant safety, it may have excluded patients with severe psychological distress, hopelessness, or suicidal ideation, which are central to the study phenomenon. This may have narrowed the range of coping experiences captured and introduced selection bias, limiting transferability to patients receiving PD with significant psychiatric comorbidity. Although patients with stable or historical psychiatric conditions were not excluded by the eligibility criteria, no such patients were recruited in the present sample. In addition, the operational definition of “other severe comorbid conditions” may have excluded patients with high comorbidity or frailty, further restricting transferability to clinically complex PD populations. Third, the study focused solely on the patients themselves and did not incorporate the perspectives of their primary caregivers, such as family members. Caregivers play a significant role in patients’ illness coping processes, and their observations and understanding of patient experiences could offer complementary insights into the phenomenon under investigation. Future research could consider employing dyadic data analysis to explore the interaction and co-constructed experiences between patients and their caregivers. Additional limitations include possible social desirability bias during face-to-face interviews, researcher influence on data generation and interpretation, potential loss of meaning in translation, predominance of urban participants, heterogeneity in PD duration, and reliance on single interviews. In addition, no formal psychological distress management or suicide-risk protocol was in place during data collection. This constitutes a methodological and ethical limitation. Finally, this study was conducted within a single cultural context. Patients’ motivations for concealing their illness and their understanding of self-transcendence may be shaped by specific Chinese cultural values, such as dignity and collectivism. The transferability of the findings to other cultural settings or healthcare systems therefore warrants caution.

## 5 Conclusion

Through in-depth interviews with 14 patients receiving peritoneal dialysis, this study explored their lived experiences and coping strategies during illness adaptation. The findings suggest that illness coping among patients receiving PD is multidimensional. Participants described interconnected illness experiences, including concealment, physical distress, and complex psychological reactions, as well as persistent daily challenges such as fixed routines, restricted mobility, and dietary limitations. They also described diverse coping strategies, including reliance on external support and self-transcendence. An in-depth exploration of illness coping experiences provides a basis for clinical nurses to identify individualized needs and formulate targeted interventions.

### 5.1 Implications for nursing practice

First, healthcare professionals should proactively explore patients’ concerns about disclosing their condition and provide emotional support to alleviate the psychological distress associated with disease-related stigma. Second, routine psychosocial screening should be integrated into follow-up care, including assessment of anxiety, depression, hopelessness, and suicide-related risk. Third, individualized support should address body-image concerns, fatigue, and mobility limitations, with practical planning for travel and daily activities. Fourth, context-sensitive dietary counseling and infection-prevention guidance should be provided, taking into account patients’ social eating practices and home environment. Fifth, structured family education should be offered to help family members participate in PD training and follow-up care. Finally, peer support groups, both online and offline, should be organized to facilitate experiential exchange and maintain social participation. Such initiatives may help patients sustain meaning and social connection while living with PD.

## Data Availability

The original contributions presented in the study are included in the article/Supplementary material, further inquiries can be directed to the corresponding authors.

## References

[ref1] Ali A. Hassiotis A. Strydom A. King M. (2012). Self stigma in people with intellectual disabilities and courtesy stigma in family carers: a systematic review. Res. Dev. Disabil. 33, 2122–2140. doi: 10.1016/j.ridd.2012.06.013, 22784823

[ref2] Bello A. K. Okpechi I. G. Osman M. A. Cho Y. Cullis B. Htay H. . (2022). Epidemiology of peritoneal dialysis outcomes. Nat. Rev. Nephrol. 18, 779–793. doi: 10.1038/s41581-022-00623-7, 36114414 PMC9483482

[ref3] Bolton L. E. Seymour J. Gardiner C. (2022). Existential suffering in the day to day lives of those living with palliative care needs arising from chronic obstructive pulmonary disease (copd): a systematic integrative literature review. Palliat. Med. 36, 567–580. doi: 10.1177/02692163221074539, 35176924 PMC9006392

[ref4] Bonomini M. Masola V. Monaco M. P. Sirolli V. Di Liberato L. Prosdocimi T. . (2024). Coupling osmotic efficacy with biocompatibility in peritoneal dialysis: a stiff challenge. Int. J. Mol. Sci. 25:3532. doi: 10.3390/ijms25063532, 38542505 PMC10971259

[ref5] Cai H. Wu Z. Xu J. Wang T. Li Y. Jiang Y. . (2024). Mitigating social isolation among peritoneal dialysis patients: the impact of educational level, physical activity, and familial support. Risk Manag Healthc Policy 17, 2271–2280. doi: 10.2147/RMHP.S479130, 39355058 PMC11444756

[ref6] Chan C. T. Wallace E. Golper T. A. Rosner M. H. Seshasai R. K. Glickman J. D. . (2019). Exploring barriers and potential solutions in home dialysis: an nkf-kdoqi conference outcomes report. Amer. J. Kidney Dis. 73, 363–371. doi: 10.1053/j.ajkd.2018.09.015, 30545707

[ref7] Cho Y. Chow K. Kam-Tao Li P. Runnegar N. Johnson D. W. (2024). Peritoneal dialysis-related infections. Clin. J. Am. Soc. Nephrol. 19, 641–649. doi: 10.2215/CJN.0000000000000280, 37574658 PMC11108252

[ref8] Cluley V. Burton J. O. Hull K. L. Eborall H. (2024). The paradox of haemodialysis: the lived experience of the clocked treatment of chronic illness. Health Sociol. Rev. 33, 24–42. doi: 10.1080/14461242.2024.2319189, 38471040

[ref9] Cohen S. Wills T. A. (1985). Stress, social support, and the buffering hypothesis. Psychol. Bull. 98, 310–357. doi: 10.1037/0033-2909.98.2.310, 3901065

[ref10] Diao K. Wang J. Huang Y. Zhang Y. Guo D. Zhang L. . (2025). The experience of social isolation in patients receiving peritoneal dialysis: a qualitative study. BMC Psychol. 13:947. doi: 10.1186/s40359-025-02367-y, 40836246 PMC12366228

[ref11] Edward K. Welch T. (2011). The extension of colaizzi's method of phenomenological enquiry. Contemp. Nurse 39, 163–171. doi: 10.5172/conu.2011.163, 22551429

[ref12] Folkman S. Moskowitz J. T. (2004). Coping: pitfalls and promise. Annu. Rev. Psychol. 55, 745–774. doi: 10.1146/annurev.psych.55.090902.141456, 14744233

[ref13] Francis J. J. Johnston M. Robertson C. Glidewell L. Entwistle V. Eccles M. P. . (2010). What is an adequate sample size? Operationalising data saturation for theory-based interview studies. Psychol. Health 25, 1229–1245. doi: 10.1080/08870440903194015, 20204937

[ref14] GBD CKDC (2020). Global, regional, and national burden of chronic kidney disease, 1990-2017: a systematic analysis for the global burden of disease study 2017. Lancet 395, 709–733. doi: 10.1016/S0140-6736(20)30045-3, 32061315 PMC7049905

[ref15] Gearing R. E. (2004). Bracketing in research: a typology. Qual. Health Res. 14, 1429–1452. doi: 10.1177/1049732304270394, 15538009

[ref16] Goffman E. (2009). Stigma: Notes on the Management of Spoiled Identity. Beijing: The Commercial Press, 137.

[ref17] Gokal R. Mallick N. P. (1999). Peritoneal dialysis. Lancet 353, 823–828. doi: 10.1016/s0140-6736(98)09410-0, 10459977

[ref18] Gomersall T. Madill A. (2015). Chronotope disruption as a sensitizing concept for understanding chronic illness narratives. Health Psychol. 34, 407–416. doi: 10.1037/hea0000151, 25197985 PMC4378531

[ref19] Griva K. Davenport A. Harrison M. Newman S. (2010). An evaluation of illness, treatment perceptions, and depression in hospital- vs. home-based dialysis modalities. J. Psychosom. Res. 69, 363–370. doi: 10.1016/j.jpsychores.2010.04.008, 20846537

[ref20] Griva K. Kang A. W. Yu Z. L. Mooppil N. K. Foo M. Chan C. M. . (2014). Quality of life and emotional distress between patients on peritoneal dialysis versus community-based hemodialysis. Qual. Life Res. 23, 57–66. doi: 10.1007/s11136-013-0431-8, 23689932

[ref21] Guedes M. Wallim L. Guetter C. R. Jiao Y. Rigodon V. Mysayphonh C. . (2022). Fatigue in incident peritoneal dialysis and mortality: a real-world side-by-side study in Brazil and the United States. PLoS One 17:e0270214. doi: 10.1371/journal.pone.0270214, 35749444 PMC9231740

[ref22] Han S. Han D. (2012). Nutrition in patients on peritoneal dialysis. Nat. Rev. Nephrol. 8, 163–175. doi: 10.1038/nrneph.2012.12, 22310948

[ref23] Himmelfarb J. Vanholder R. Mehrotra R. Tonelli M. (2020). The current and future landscape of dialysis. Nat. Rev. Nephrol. 16, 573–585. doi: 10.1038/s41581-020-0315-4, 32733095 PMC7391926

[ref24] Hossain S. Anjum A. Hasan M. T. Uddin M. E. Hossain M. S. Sikder M. T. (2020). Self-perception of physical health conditions and its association with depression and anxiety among bangladeshi university students. J. Affect. Disord. 263, 282–288. doi: 10.1016/j.jad.2019.11.153, 31818790

[ref25] Jung H. Jeon Y. Park Y. Kim Y. S. Kang S. Yang C. W. . (2019). Better quality of life of peritoneal dialysis compared to hemodialysis over a two-year period after dialysis initiation. Sci. Rep. 9:10266. doi: 10.1038/s41598-019-46744-1, 31312004 PMC6635359

[ref26] Kalantar-Zadeh K. Li P. K. Tantisattamo E. Kumaraswami L. Liakopoulos V. Lui S. . (2021). Living well with kidney disease by patient and care partner empowerment: kidney health for everyone everywhere. Transpl. Int. 34, 391–397. doi: 10.1111/tri.13811 33382133

[ref27] Lev-Wiesel R. Sasson L. Scharf N. Abu Saleh Y. Glikman A. Hazan D. . (2022). "losing faith in my body": body image in individuals diagnosed with end-stage renal disease as reflected in drawings and narratives. Int. J. Environ. Res. Public Health 19:10777. doi: 10.3390/ijerph191710777, 36078494 PMC9517917

[ref28] Liu X. Xu Q. Ma X. Xu Y. (2025). A pathway analysis of factors affecting quality of life in peritoneal dialysis patients: a cross-sectional study based on individual and family self-management theory. Patient Prefer. Adherence 19, 3527–3540. doi: 10.2147/PPA.S553320, 41230338 PMC12604591

[ref29] Livingston J. D. Boyd J. E. (2010). Correlates and consequences of internalized stigma for people living with mental illness: a systematic review and meta-analysis. Soc. Sci. Med. 71, 2150–2161. doi: 10.1016/j.socscimed.2010.09.030 21051128

[ref30] Lv J. Zhang L. (2019). Prevalence and disease burden of chronic kidney disease. Adv. Exp. Med. Biol. 1165, 3–15. doi: 10.1007/978-981-13-8871-2_1, 31399958

[ref31] Madeira L. Leal B. Filipe T. Rodrigues M. F. Figueira M. L. (2019). The uncanny of the illness experience: can phenomenology help? Psychopathology 52, 275–282. doi: 10.1159/000504141, 31722355

[ref32] Malli M. A. Forrester-Jones R. Murphy G. (2016). Stigma in youth with tourette's syndrome: a systematic review and synthesis. Eur. Child Adolesc. Psychiatry 25, 127–139. doi: 10.1007/s00787-015-0761-x, 26316059

[ref33] Marki E. Moisoglou I. Aggelidou S. Malliarou M. Tsaras K. Papathanasiou I. V. (2023). Body image, emotional intelligence and quality of life in peritoneal dialysis patients. Aims Public Health 10, 698–709. doi: 10.3934/publichealth.2023048, 37842280 PMC10567979

[ref34] Park C. L. (2010). Making sense of the meaning literature: an integrative review of meaning making and its effects on adjustment to stressful life events. Psychol. Bull. 136, 257–301. doi: 10.1037/a0018301, 20192563

[ref35] Reed P. G. (1991). Toward a nursing theory of self-transcendence: deductive reformulation using developmental theories. ANS Adv. Nurs. Sci. 13, 64–77. doi: 10.1097/00012272-199106000-00008, 2059006

[ref36] Sandhu A. Ives J. Birchwood M. Upthegrove R. (2013). The subjective experience and phenomenology of depression following first episode psychosis: a qualitative study using photo-elicitation. J. Affect. Disord. 149, 166–174. doi: 10.1016/j.jad.2013.01.018, 23414936

[ref37] Sharpe L. Michalowski M. Richmond B. Menzies R. E. Shaw J. (2023). Fear of progression in chronic illnesses other than cancer: a systematic review and meta-analysis of a transdiagnostic construct. Health Psychol. Rev. 17, 301–320. doi: 10.1080/17437199.2022.2039744, 35132937

[ref38] Shilling C. (2021). The Body and Social Theory. Shanghai: Shanghai Literature and Art Publishing House, 3.

[ref39] Silva C. G. D. Crossetti M. D. G. A. Fernández M. G. Prates J. D. S. (2024). Middle-range theory of the existential dimension of being-in-the-world of chronic kidney disease: grounded theory. Rev. Bras. Enferm. 77:e20230152. doi: 10.1590/0034-7167-2023-0152, 39319965 PMC11419687

[ref40] Tan J. H. Sharpe L. Russell H. (2021). The impact of ovarian cancer on individuals and their caregivers: a qualitative analysis. Psychooncology 30, 212–220. doi: 10.1002/pon.5551, 32940943

[ref41] Tedeschi R. G. (2023). The post-traumatic growth approach to psychological trauma. World Psychiatry 22, 328–329. doi: 10.1002/wps.21093, 37159363 PMC10168173

[ref42] Tong A. Sainsbury P. Craig J. (2007). Consolidated criteria for reporting qualitative research (coreq): a 32-item checklist for interviews and focus groups. Int. J. Qual. Health Care 19, 349–357. doi: 10.1093/intqhc/mzm042, 17872937

[ref43] Wilkinson T. J. Nixon D. G. D. Palmer J. Lightfoot C. J. Smith A. C. (2021). Differences in physical symptoms between those with and without kidney disease: a comparative study across disease stages in a Uk population. BMC Nephrol. 22:147. doi: 10.1186/s12882-021-02355-5, 33888089 PMC8063370

[ref44] Wu X. Zhang X. Li X. Huang G. Chen X. Gong T. (2026). Coping experiences of patients with moderate to severe burns: a descriptive qualitative study. J. Adv. Nurs. 82, 5122–5131. doi: 10.1111/jan.70145, 40817704

[ref45] Xia W. Ou M. Chen Y. Chen F. Yan M. Xiao Z. . (2024). Experiences of patients with advanced cancer coping with chronic pain: a qualitative analysis. BMC Palliat. Care 23:94. doi: 10.1186/s12904-024-01418-2, 38600476 PMC11005139

[ref46] Xiong W. An T. (2025). Resilience and adaptive strategies for managing stigma in chinese people with chronic kidney disease. Sci. Rep. 15:26721. doi: 10.1038/s41598-025-12676-2, 40702230 PMC12287454

[ref47] Yang X. Shan Y. Gao Y. Wang H. Li X. Ding Y. . (2024). Inner strength, coping self-efficacy and coping strategy of patients with peritoneal dialysis: a exploratory cross-sectional study. Nurs. Open 11:e2127. doi: 10.1002/nop2.2127, 39219185 PMC11366776

[ref48] Yi C. Wang X. Ye H. Lin J. Yang X. (2022). Patient-reported gastrointestinal symptoms in patients with peritoneal dialysis: the prevalence, influence factors and association with quality of life. BMC Nephrol. 23:99. doi: 10.1186/s12882-022-02723-9, 35264119 PMC8905857

[ref49] Yngman-Uhlin P. Kjellsdotter A. Uhlin F. Edéll-Gustafsson U. (2019). Sleep quality, fatigue, and health-related quality of life in patients on initial peritoneal dialysis and multiple modalities after two years: a prospective study. Nephrol. Nurs. J. 46, 615–649. 31872992

[ref50] Zugaj M. R. Busch C. Züger A. Ke Ler J. (2025). Experience of illness with chronic singultus: a qualitative interview study. Orphanet J. Rare Dis. 20:141. doi: 10.1186/s13023-025-03619-1 PMC11930004 40121512

