# Supplementary material for: Illness coping experiences among patients receiving peritoneal dialysis: a descriptive phenomenological study
Source: Front Psychol. 2026 Oct 28;17:1924070. doi: 10.3389/fpsyg.2026.1924070 (PMC13752181; doi:10.3389/fpsyg.2026.1924070)
Supplement: Supplementary file 1 [file Supplementary_file_1.docx]

# S1 Checklist.

**Consolidated criteria for reporting qualitative studies (COREQ): 32-item checklist**

A checklist of items that should be included in reports of qualitative research. You must report the page number in your manuscript where you consider each of the items listed in this checklist. If you have not included this information, either revise your manuscript accordingly before submitting or note N/A.

| **No. Item** | **Guide questions/description** | **Reported on Page #/Response** |
| --- | --- | --- |
| **Domain 1: Research team and reﬂexivity** |  |  |
| *Personal Characteristics* |  |  |
| 1. Interviewer/ facilitator | Which author/s conducted the interview or focus group? | Methods – Design |
| 2. Credentials | What were the researcher’s credentials? E.g. PhD, MD | Not reported |
| 3. Occupation | What was their occupation at the time of the study? | Methods – Design |
| 4. Gender | Was the researcher male or female? | Not reported |
| 5. Experience and training | What experience or training did the researcher have? | Methods – Design |
| *Relationship with*  *participants* |  |  |
| 6. Relationship established | Was a relationship established prior to study commencement? | Methods – Design |
| 7. Participant knowledge of the interviewer | What did the participants know about the researcher? e.g. personal goals, reasons for doing the research? | Methods-Study Setting and Recruitment |
| 8. Interviewer characteristics | What characteristics were reported about the interviewer/facilitator? e.g. Bias, assumptions, reasons and interests in the research topic | Methods – Design；Methods-Data Collection |
| **Domain 2: study**  **design** |  |  |
| *Theoretical framework* |  |  |
| 9. Methodological orientation and Theory | What methodological orientation was stated to underpin the study? e.g. grounded theory, discourse analysis, ethnography, phenomenology, content analysis | Methods – Design |

| *Participant selection* |  |  |
| --- | --- | --- |
| 10. Sampling | How were participants selected? e.g. purposive, convenience, consecutive, snowball | Methods-Study Setting and Recruitment |
| 11. Method of  approach | How were participants approached? e.g. face-to-  face, telephone, mail, email | Methods – Design |
| 12. Sample size | How many participants were in the study? | Methods-Study Setting and Recruitment |
| 13. Non-participation | How many people refused to participate or dropd out? Reasons? | Not reported |
| *Setting* |  |  |
| 14. Setting of data collection | Where was the data collected? e.g. home, clinic, workplace | Methods-Data Collection |
| 15. Presence of non- participants | Was anyone else present besides the participants and researchers? | Methods-Data Collection |
| 16. Description of sample | What are the important characteristics of the sample? e.g. demographic data, date | Methods – Study setting and recruitment; Findings |
| *Data collection* |  |  |
| 17. Interview guide | Were questions, prompts, guides provided by the authors? Was it pilot tested? | Methods-Data Collection |

| 18. Repeat interviews | Were repeat interviews carried out? If yes, how  many? | Not reported |
| --- | --- | --- |
| 19. Audio/visual recording | Did the research use audio or visual recording to collect the data? | Methods-Data Collection |
| 20. Field notes | Were ﬁeld notes made during and/or after the interview or focus group? | Methods-Data Collection |
| 21. Duration | What was the duration of the interviews or focus group? | Findings-Themes |
| 22. Data saturation | Was data saturation discussed? | Methods – Study setting and recruitment |
| 23. Transcripts returned | Were transcripts returned to participants for comment and/or correction? | Methods-Data Analysis |
| **Domain 3: analysis and ﬁndings** |  |  |
| *Data analysis* |  |  |
| 24. Number of data coders | How many data coders coded the data? | Methods-Data Analysis |
| 25. Description of the  coding tree | Did authors provide a description of the coding  tree? | Not reported |
| 26. Derivation of themes | Were themes identiﬁed in advance or derived from the data? | Methods-Data Collection |
| 27. Software | What software, if applicable, was used to manage the data? | Not reported |
| 28. Participant checking | Did participants provide feedback on the ﬁndings? | Methods-Data Analysis |
| *Reporting* |  |  |
| 29. Quotations presented | Were participant quotations presented to illustrate the themes/ﬁndings? Was each quotation identiﬁed? e.g. participant number | Findings |
| 30. Data and ﬁndings  consistent | Was there consistency between the data  presented and the ﬁndings? | Findings; Discussion |

| 31. Clarity of major themes | Were major themes clearly presented in the ﬁndings? | Findings – Themes |
| --- | --- | --- |
| 32. Clarity of minor themes | Is there a description of diverse cases or discussion of minor themes? | Findings; Discussion |

Once you have completed this checklist, please save a copy and upload it as part of your submission. When requested to do so as part of the upload process, please select the file type: *Checklist*. You will NOT be able to proceed with submission unless the checklist has been uploaded. Please DO NOT include this checklist as part of the main manuscript document. It must be uploaded as a separate file.
