# Supplementary material for: Illness coping experiences among patients receiving peritoneal dialysis: a descriptive phenomenological study
Source: Front Psychol. 2026 Oct 28;17:1924070. doi: 10.3389/fpsyg.2026.1924070 (PMC13752181; doi:10.3389/fpsyg.2026.1924070)
Supplement: Supplementary file 2 [file Supplementary_file_2.docx]

## Illustrative example of the analytic progression

| Step | Content |
| --- | --- |
| Raw quotation (translated from Chinese) | Being on PD isn't exactly a good thing. I was worried that if other people found out, word would get around. There'd be gossip, people pointing fingers at me. So only my family and a few close friends know. I've never brought it up with anyone else. |
| Significant statement | The participant concealed the illness from others because she feared gossip and social judgement. |
| Formulated code | Concealment to avoid gossip and social judgement. |
| Subtheme | Concealment of Illness. |
| Final theme | Illness Experiences. |
